# Graph-based prediction of reaction barrier heights with on-the-fly prediction of transition states

**DOI:** 10.1039/d5dd00240k

**Published:** 2025-09-15

**Authors:** Johannes Karwounopoulos, Jasper De Landsheere, Leonard Galustian, Tobias Jechtl, Esther Heid

**Affiliations:** a Institute of Materials Chemistry, TU Wien A-1060 Vienna Austria esther.heid@tuwien.ac.at

## Abstract

The accurate prediction of reaction barrier heights is crucial for understanding chemical reactivity and guiding reaction design. Recent advances in machine learning (ML) models, particularly graph neural networks, have shown great promise in capturing complex chemical interactions. Here, directed message-passing neural networks (D-MPNNs) on graph overlays of the reactant and product structures were shown to provide promising accuracies for reaction property prediction. They rely solely on molecular graph changes as input and thus require no additional information during inference. However, the reaction barrier height intrinsically depends on the conformations of the reactants, transition state, and products, which are not taken into account in standard D-MPNNs. In this work, we present a hybrid approach where we combine the power of D-MPNNs predicting barrier heights with generative models predicting transition state geometries on-the-fly for organic reactions. The resulting model thus only requires two-dimensional graph information as input, while internally leveraging three-dimensional information to increase accuracy. We furthermore evaluate the influence of additional physical features on D-MPNN models of reaction barrier heights, where we find that additional features only marginally enhance predictive accuracy and are especially helpful for small datasets. In contrast, our hybrid graph/coordinate approach reduces the error of barrier height predictions for the two investigated datasets RDB7 and RGD1.

## Abstract 2

The accurate prediction of reaction barrier heights is crucial for understanding chemical reactivity and guiding reaction design.

## Graphical abstract

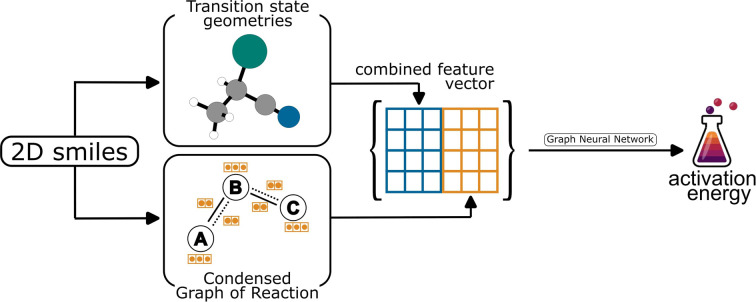

## 1 Introduction

In computational chemistry, accurate knowledge of reaction barrier heights (or activation energies) is crucial for understanding chemical reactions.^1^ However, obtaining these barriers computationally requires high-level quantum mechanical (QM) calculations, which are computationally expensive and scale poorly with system size. Recent advances in machine learning offer a promising alternative: graph neural networks (GNNs) trained on QM-calculated data can rapidly predict reaction barrier heights at a fraction of the computational cost while maintaining predictive accuracy comparable to high-level quantum methods.^2–6^ To enable such predictions, the choice of representation plays a central role in how well a machine learning model can capture the underlying chemistry.

For example, Grambow *et al.*^7^ adapted a directed message passing neural network (D-MPNN) originally implemented for molecular property prediction^8,9^ to encode separate graph representations of reactants and products, and to predict reaction barrier heights based on the differences in encodings for each atom. However, its good performance was later shown to arise from data leakage to the test set, whereas the actual predictive performance on new datapoints was rather poor.^10^ Heid and Green^10^ significantly improved on the accuracy of barrier height predictions using D-MPNNs by representing reactions as a single graph, the condensed graph of reaction (CGR), which is constructed by superimposing reactant and product graphs. This model was later refined using extensive pretraining and reaction enthalpies as additional features, as well as additional atom and bond features such as ring sizes.^11^

Subsequent studies focused on integrating further chemically meaningful descriptors, both expert-curated or computationally derived, to better capture reaction-specific characteristics like electron distribution, transition state geometry, or local reactivity environments.^12–16^ In line with this trend, Stuyver and Coley^17^ used a hybrid QM-augmented model architecture that first predicts a set of atom and bond level descriptors derived from density functional theory (DFT) calculations and integrated them into a model to predict activation energies.^17^ Vargas *et al.*^18^ enhanced predictive accuracy by incorporating electronic density-based descriptors derived from the quantum theory of atoms in molecules (QTAIM), leading to a substantial increase in accuracy.^19^ In a related approach, García-Andrade *et al.*^20^ augmented their model with the full set of molecular descriptors available from RDKit. However, a feature importance analysis indicated that the majority of these features contributed little to the model's performance.^20^ Other approach combined low-cost semi-empirical quantum mechanical (SQM) methods with machine learning methods, where the SQM calculations were used to generate geometries and energies, which were then integrated into the model.^4,21–23^

While the above approaches mainly rely on 2D molecular representations, it is important to recognise that reaction pathways are inherently three-dimensional. Different spatial arrangements can lead to varying barrier heights, meaning that 2D-based models are ultimately limited by aleatoric uncertainty introduced by unaccounted 3D structural information. Recent work has explored the integration of three-dimensional structural information directly into graph neural network (GNN) architectures.^24,25^ For reaction barrier height predictions, van Gerwen *et al.*^26^ developed 3DReact, a model that incorporates the spatial information of reactants and products within a GNN framework.^26^

Yet the relevant information lies in the transition state (TS), not the reactant and product information. The TS represents the highest energy structure along the reaction pathway at the top of the energy barrier between reactants and products. Thus, it is crucial for determining the activation energy, which is calculated from the energy difference between the reactant and the TS. However, incorporating 3D information necessitates access to accurate molecular and TS geometries, which poses a significant limitation as they need to be provided from QM calculations. Once a TS geometry is obtained computationally, its energy is also known, making a machine-learning-based prediction of the barrier height obsolete. However, TS geometries themselves can be predicted using neural networks,^27,28^ providing a basis for calculating activation energies from the energy difference between the TS structure and the reactant directly. Kim *et al.*^29^ recently introduced TSDiff, a generative diffusion model that predicts TS geometries directly from SMILES strings. By modeling the distribution of TS structures through a stochastic diffusion process, TSDiff can generate geometries without the need for precomputed 3D information of the reactant or product. Galustian *et al.*^30^ further proposed GoFlow, which combines flow matching with E(3)-equivariant neural networks to generate coordinates. Similarly to TSDiff, GoFlow only needs 2D molecular graphs as input, but is significantly more efficient than a diffusion-based model. These developments open the possibility of generating high-quality 3D structural data on-the-fly. In this work, we use the positional information generated by TSDiff and GoFlow and integrate it into a D-MPNN framework to predict accurate activation barriers, relying only on 2D representations while implicitly capturing critical 3D structural insights.

## 2 Methods

All our experiments are conducted using ChemTorch,^31^ which is an open-source framework for developing and benchmarking chemical reaction property prediction models.

### 2.1 Feature vectors in the condensed graph of reaction

**Fig. 1:**
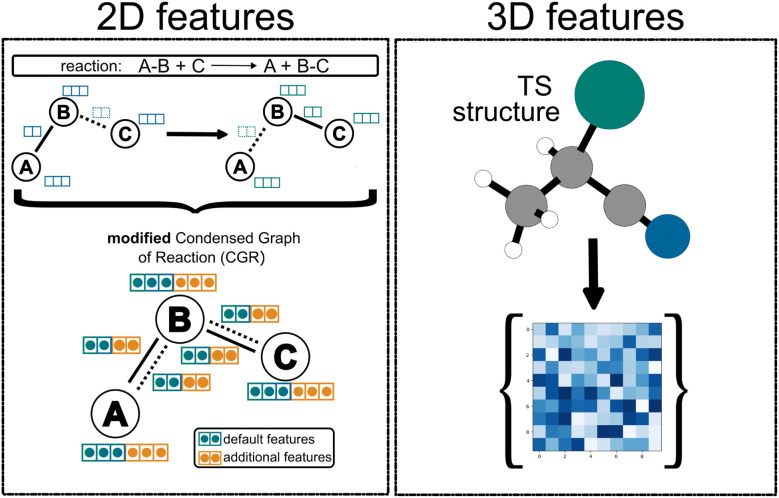
Left: CGR as a graph overlay of reactants and products, showing the condensed feature vector for each atom and bond in blue; and additional features which can be attached to the initial CGR features by a simple concatenation in orange. Right: descriptors are calculated for the TS structures and concatenated with the original feature vector.

In the framework of graph neural networks, molecules are described as graphs, where nodes *V* correspond to atoms and edges *E* represent chemical bonds. For each atom (node) *v*, atom feature vectors {*x*_*v*_∣*v* ∈ *V*} are computed using basic RDKit properties, namely the atomic number, the number of bonds linked to each atom, the formal charge, the hybridization, the number of hydrogens, the aromaticity of the atom, as well as the atomic mass (divided by 100 for scaling). For each bond (edge) between the atoms *v* and *w* a feature vector {*e*_*vw*_∣{*v*, *w*} ∈ *E*} is constructed from the bond order (single, double, triple or aromatic), whether the bond is conjugated and whether the bond is part of a ring system and if so of which ring size. These default features are used as suggested in the chemprop implementation.^9^ To create directed edges, atom features are then concatenated with bond features *via e*_*vw*_ = cat(*x*_*v*_, *e*_*vw*_). To encode a reaction, the graphs of the reactants and products are then superimposed into a single reaction graph, the CGR (Fig. 1)^10,32,33^ and the atom and bond features are concatenated. This approach ensures that modifications to bond connectivity, such as bond formation, are explicitly reflected in the graph structure and the bond features. For example, if a bond between atoms A and B is present in the reactant but absent in the product, the encoding retains this distinction. In this work, we include additional features into the CGR framework as either atom, bond, or molecular features by concatenating them with the original CGR feature vectors, as shown in Fig. 1, top left.

### 2.2 Directed message-passing neural network

The atom and bond features, as introduced in the previous section, are processed by a D-MPNN.^8,9^ Hidden directed edge features *h*_*vw*_^0^ with dimensionality *h* are created by passing the directed edge features *e*_*vw*_ through a linear layer. These initial directed edge features are then iteratively updated using message passing over *T* steps:1
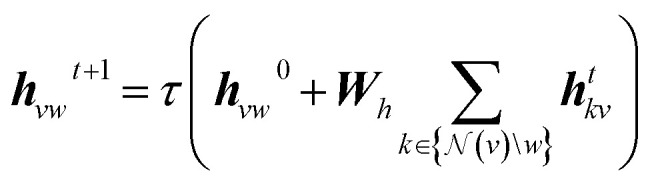
Here, ***W***_*h*_ ∈ ***R***^*h*×*h*^ is a learnable weight matrix, and 
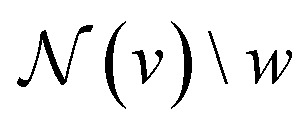
 denotes the set of neighbours of node *v* excluding node *w*. The summation aggregates messages from the local incoming edges. After the final message passing step, the directed edge hidden states ***h***^*T*^_*vw*_ are aggregated to obtain atom-level embeddings *h*_*v*_ using:

2 ***h***_*v*_ = *τ*(***W***_*0*_***q***)

where ***q*** is the concatenation of the initial atom features *x*_*v*_ and the sum of incoming directed edge hidden states. Once the atomic embeddings ***h***_*v*_ are computed for all atoms, they are aggregated into a single molecular representation ***h***_*m*_ using a pooling function:3
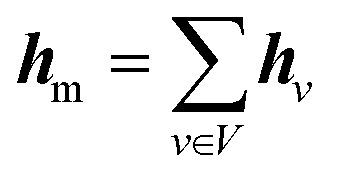


Finally, activation energies are predicted from the molecular embeddings ***h***_m_ using a feed-forward neural network (FFN).

### 2.3 Prediction of 2D features

Li *et al.*^34^ recently introduced a D-MPNN to predict atom, bond, and molecular descriptors trained on quantum mechanical information, which we refer to as machine-learned quantum mechanical (ml-QM) descriptors. We integrate the ml-QM descriptors into our reaction D-MPNN as additional features (orange vectors in Fig. 1).

For constructing their model, Li *et al.*^34^ computed 37 quantum mechanical (QM) descriptors across atomic, bond, and molecular levels for a large dataset. The 37 descriptors include 13 atom-level properties (*e.g.*, NPA charges, Parr functions, NMR shielding constants, valence orbital occupancies), 4 bond-level features (*e.g.*, bond order, bond length, bonding electrons, natural ionicity), and 20 molecular-level descriptors (*e.g.*, energy gaps, ionization potential, electron affinity, dipole and quadrupole moments), as detailed in Table S2.

To support on-the-fly inference of ml-QM features, Li *et al.*^34^ provide three specialized models: one model focuses on atom and bond properties, another on molecular dipole and quadrupole moment predictions, and the third on energy gaps, ionisation potential, and electron affinity.^34^ We utilized the pretrained models as provided in the literature.^34^ As these models were specifically designed to predict features for individual molecules, we split the reaction SMILES into reactant and product components and computed the corresponding ml-QM properties for each part separately.

### 2.4 3D features

To incorporate 3D features into graph-based models, we need to encode 3D local environments around each atom into vectors (described in Section 2.4.1), embed them into the D-MPNN (described in Section 2.4.2), and learn the actual 3D coordinates using a generative model (described in Section 2.4.3).

#### 2.4.1 Different descriptors for positional encoding

Coordinate information was integrated into the model using local descriptors commonly employed in machine-learned force fields, where capturing 3D structural information is a key aspect.^35^ For each compound in a reaction (reactant, transition state, and product), we calculated the respective descriptor vector and concatenated them. We evaluated four widely used descriptors. First, we used two simpler approaches of fixed descriptors which are obtained from an analytic function: the SOAP descriptor^36^ which encodes coordinates of molecules through a smooth overlap of atomic positions (SOAP) vector and the Atomic Environment Vector (AEV) as used in the ANI and AIMNet models which uses modified Behler and Parrinello symmetry functions.^37^ We then explored two learned descriptors, extracted from the hidden atomic representations of machine-learned foundation models. The multi-Atomic Cluster Expansion descriptor was used as implemented in the MACE architecture.^38,39^ As pretrained model we used the MACE-MP0 and the MACE-OFF medium model.^40,41^ Further, we computed hidden atomic representations of the Equiformer V2 architecture.^42^ Here we used the eqv2_31m_omat model as the pretrained model.^43^ The obtained descriptors were treated as additional atomic features and concatenated with the default features, as described in Section 2.1.

#### 2.4.2 Embedding of positional feature vectors

**Fig. 2:**
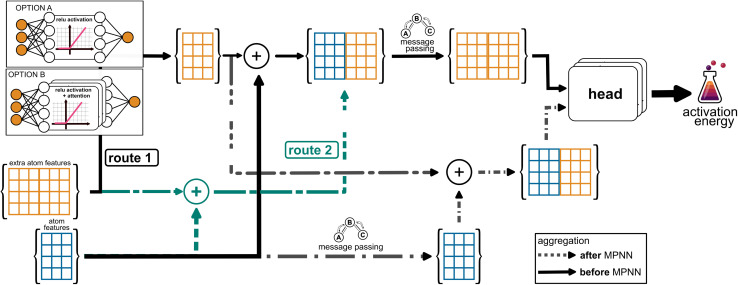
Possible approaches for integrating additional atomic features into the D-MPNN. In Route 1, the extra atomic feature vector is first processed through a linear layer with a ReLU activation function (Option 1) or through a linear layer with a ReLU activation followed by an additional attention layer (Option 2). The transformed vector is then concatenated with the original atomic feature vector. In Route 2, the extra atomic feature vector is directly concatenated with the original atomic features without prior transformation. In both cases, the concatenation can occur either before the message-passing step (solid black and dashed green lines) or afterwards (dashed black lines).

Due to the high dimensionality of the feature vectors obtained from the coordinate information, we reduced the dimensionality of the vectors by processing the initial features with an additional layer to extract the most relevant information. Fig. 2 illustrates two main approaches for incorporating atomic features derived from 3D coordinates into the D-MPNN. In Route 1, the extra atomic feature vector undergoes a processing step to learn a meaningful representation of the positional feature vector. This transformation is achieved through a linear layer with a ReLU activation function (Option 1) or a linear layer followed by a ReLU activation and an additional attention layer (Option 2). Subsequently, the processed atomic feature vector is concatenated with the original atomic feature vector. In Route 2, the extra atomic feature vector is directly concatenated with the original atomic features without any prior processing. Both Route 1 and Route 2 can occur either before the message-passing step (solid black and dashed green lines) or after the D-MPNN process (dashed black lines). In the former case, the model has the opportunity to propagate the atomic features through the graph network. In the latter case, the processed atomic features are incorporated only in the final prediction step, which may help in reducing feature redundancy.

#### 2.4.3 Generative models to predict geometries on-the-fly

##### 2.4.3.1 Diffusion model

We used TSDiff^29^ as the diffusion model to generate the 3D coordinates of transition states of each reaction. TSDiff was trained using 5000 sampling steps during inference and all configuration parameters as recommended in the original literature by Kim *et al.*^29^.

##### 2.4.3.2 Flow matching model

GoFlow was used as provided in the literature^30^ to generate 3D coordinates of the transition states of each reaction. Following the recommended setup, we used the default configuration with 25 ODE steps and 25 samples during inference. For consistency, we applied the same train/validation split as used in the diffusion model.

### 2.5 Datasets

To apply ml-QM features, we utilized the RDB7, RGD1, RDB19-Rad, Cycloaddition, and E_2_Sn_2_ datasets as detailed below. We ensured that all reactions were balanced and included explicit hydrogens. We randomly split each dataset into train, validation and test set (90%/5%/5%). For the RDB7 and RGD1 datasets that contain forward and reverse reactions, we only used the forward reaction. For the work with positional information, we only used the RDB7 and RGD1 datasets.

#### 2.5.1 RDB7

The RDB7 dataset^44^ builds upon the work of Grambow *et al.*^45^. The geometries of 11 926 compounds were optimized using the *ω*B97X-D3/def2-TZVP level of theory, with single-point energies refined at the CCSD(T)-F12a/cc-pVDZ-F12 level for the actual barrier heights. The data set encompasses a wide variety of reaction types, involving up to seven heavy atoms (C, H, N, and O).

#### 2.5.2 RGD1

The Reaction Graph Depth 1 (RGD1) dataset^46^ comprises 176 992 organic reactions with up to 10 heavy (non-hydrogen) atoms, including C, O, N and H atoms. Transition states were identified using the growing string method at a semi-empirical level of theory and subsequently validated with higher-level DFT calculations (B3LYP-D3/TZVP). Reactant and product geometries were also optimised at the B3LYP-D3/TZVP level. The data set predominantly features b2f2 reactions, which involve ”breaking two bonds and forming two bonds”.

#### 2.5.3 RDB19-rad

The RDB19-rad dataset^47^ contains 5600 radical reactions in different solvents with the elements H, C, N, O, and S, and contains up to 19 heavy atoms. Geometries were optimised at the B3LYP-D3(BJ)/def2-TZVP level of theory. Single-point energies for the barrier height calculations were calculated using the M06-2X functional with the def2-QZVP basis set. We extracted the barrier heights from the gas-phase calculations.

#### 2.5.4 Cycloaddition

The cycloaddition dataset^48^ focuses on computational profiles for [3 + 2] dipolar cycloaddition reactions, involving molecules composed of C, H, O, and N atoms for a total of 5269 reactions. Geometry optimizations and frequency calculations were conducted using B3LYP-D3(BJ)/def2-SVP, with single-point energy refinements at B3LYP-D3(BJ)/def2-TZVP, incorporating implicit solvation with the SMD model for the calculation of the activation barrier heights.

#### 2.5.5 E_2_Sn_2_

The E_2_Sn_2_ dataset^49^ provides barrier heights for E_2_ (elimination) and Sn_2_ (substitution nucleophilic) reaction pathways for 3626 reactions. It covers a diverse chemical combination of substituents, nucleophiles (*e.g.*, H, F, Cl, Br), and leaving groups. Geometry optimizations were conducted at the MP2/6-311G(d) level of theory. Barrier heights were obtained from refined single-point energy calculations using DF-LCCSD/cc-pVTZ. SMILES strings were used as provided in ref. 10.

#### 2.5.6 Heterocycles

Radical C–H bond functionalization reactions involving heterocyclic compounds, comprising data for 6114 molecules.^50^

#### 2.5.7 Nitroaddition

A dataset of 5269 nitro-Michael cycloaddition reactions, including transition state (TS) structures and reaction barriers calculated using semi-empirical quantum mechanical (SQM) methods and refined *via* machine learning (ML) to approximate DFT-level accuracy.^22^

### 2.6 Hyperparameters

All experiments were conducted on an NVIDIA A40, which has 48 GB of GPU memory. The following hyperparameters were kept constant across all model trainings. We employed the AdamW optimizer^51^ with default parameters: *β*_1_ = 0.9, *β*_2_ = 0.999, and *ε* = 10^−8^. As a scheduler, we used cosine annealing with 10 steps for the warmup phase. We used a batch size of 50 and trained each network for 100 epochs.

Hyperparameter tuning was performed using Bayesian optimization, aiming to minimise the validation MAE. We varied the learning rate, the dropout rates, the number of hidden channels in both the message-passing network and the prediction head, the depth of the message-passing network, and the number of layers in the prediction head. The specific values used for each parameter are listed in Table S1. Hyperparameter optimization was run only on the RDB7 dataset, as it offers a well-balanced mix of diverse reactions at a moderate dataset size.

### 2.7 Different splitting strategies

For all studies on finding the best architecture for incorporating additional features, we use a heuristic random split, randomly assigning reactions to the training, validation, or test set. To assess our models' generalizability, we also employ two specialized splits: reaction core and barrier height. For the reaction core split, we extract the reaction core of each reaction and cluster them by their common core. All reactions belonging to the same common core are randomly assigned to either the training, validation, or reaction set. For the barrier height split, we collect the upper and lower 5% with the highest and lowest barrier height and create the test and validation set from that, while the 90% in between serves as the training set.

## 3 Results and discussion

### 3.1 Evaluating the influence of ml-QM features

The results presented in Table 1 compare the predictive performance when using default features *versus* using default plus additional ml-QM features for different datasets. Across all datasets, the inclusion of additional features leads to a modest improvement in predictive accuracy. For instance, in the E_2_ dataset, MAE decreases from 2.51 to 2.43, and RMSE improves from 3.75 to 3.64. Similarly, the RDB7 dataset shows a slight decrease in the RMSE (6.68 to 6.24). However, in the RGD1 dataset, the improvement is marginal, indicating that additional ml-QM features do not significantly impact the performance in this case.

**Table 1:** Mean Absolute Error (MAE) and Root Mean Square Error (RMSE) for different datasets using default features and additional ml-QM features

Dataset	Default features		Additional ml-QM features	
	MAE	RMSE	MAE	RMSE
E_2_	2.51 ± 0.08	3.75 ± 0.09	2.43 ± 0.10	3.64 ± 0.05
RDB7	3.58 ± 0.08	6.68 ± 0.13	3.38 ± 0.07	6.24 ± 0.18
RGD1	4.02 ± 0.02	6.91 ± 0.02	3.99 ± 0.05	7.08 ± 0.05

To furthermore assess the importance of ml-QM features, we conducted a permutation feature analysis, where each feature was permuted individually during prediction.^52^ An increase in MAE indicates that the permutated feature was essential for the model, as its permutation disrupted the predictions. Conversely, a decrease in MAE suggested that the feature negatively impacted the model's performance. As illustrated in Fig. S1, permuting the molecular features had no noticeable effect on the prediction accuracy. In contrast, several atomic and bond features exhibited a stronger influence, particularly the NPA charges and the 2p valence orbital occupancy. Across three datasets (E_2_, RDB7, and RGD1), the same features consistently ranked among the top ten, albeit in varying orders. We examined the model performance for the RDB7 dataset using only the ten most important features. This resulted in an MAE of 3.42 and an RMSE of 6.06, comparable to the MAE when utilising the full feature set (see Table 1).

**Fig. 3:**
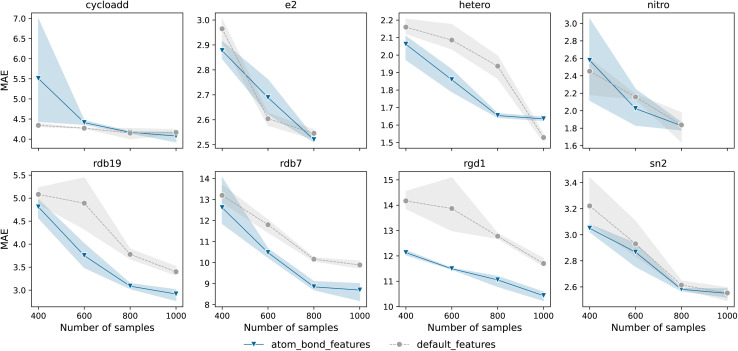
Test MAE of models trained on randomly split training sets of different sizes, sampled from the seven datasets outlined in the Methods section. The dots represent the average values computed over three runs with different random seeds, while the shaded region indicates the error bars. Results obtained using the default features are shown in grey, and those incorporating additional atom and bond features are represented in blue. The MAE is in kcal mol^−1^.

As observed in the literature on molecular property prediction, where additional ml-QM features are particularly beneficial for smaller datasets,^34^ we investigated this effect by artificially reducing the size of our datasets, selecting only a fraction of the available data. As illustrated in Fig. 3, this trend holds true for most datasets analysed in this study. Incorporating ml-QM features alongside the default features significantly lowers the test MAE, with the effect being especially pronounced in diverse datasets such as RDB7 and RGD1. However, for simpler datasets that focus on a single reaction type, such as cycloaddition or S_N_2, the inclusion of additional ml-QM features does not appear to improve the model's predictive accuracy.

As indicated by the feature importance analysis, the incorporation of molecular features is not beneficial for the model and does not improve the results, as shown in Fig. S2.

### 3.2 3D features

We used the RDB7 and RGD1 datasets to include 3D information into our D-MPNN model. We refer to the coordinates recorded in the datasets as the ground truth coordinates. First, we used only the RDB7 dataset as a model case, as it provides a good balance between computational efficiency and dataset size as well as complexity (including multiple reaction types). In the first section, we test the different descriptors (Section 3.2.1). We then continue using the best performing descriptor for evaluating which positional information is most beneficial, testing different combinations of reactant, transition state and/or product positions (Section 3.2.2). For the best-performing combination of descriptor and positional information, we test different ways of embedding this information (Section 3.2.3) into the model. Finally, we combine all these findings and use the best-performing combination of all these parameters to build models with on-the-fly 3D coordinate prediction for both RDB7 and RGD1.

#### 3.2.1 Testing different descriptors for incorporating coordinate information

First, we test the different proposed descriptors by adding them as plain additional features before the message-passing step (see also Fig. 2). The MAE and RMSE values are shown in Table 2. The SOAP descriptor^36^ yields a MAE of 4.74 and a RMSE of 6.99. The aniAEV descriptor^37^ improves performance slightly, achieving an MAE of 3.93 and an RMSE of 6.09. The descriptor provided by the Equiformer V2 architecture,^42^ tested in combination with the OMAT model, achieves a MAE of 3.21 and a RMSE of 4.50. Among the models tested, the MACE descriptors^38,39^ demonstrated the best overall performance. Combined with the mace-mp0 foundation model, an MAE of 2.44 and an RMSE of 4.09 can be achieved, while the mace-off variant provides slightly less accuracy with an MAE of 2.46 and an RMSE of 4.17.

**Table 2:** MAE and RMSE for the five descriptors tested. For each descriptor, a hyperparameter search using Bayesian optimization with at least 200 individual runs was performed. All values are in kcal mol^−1^

Architecture	Model	MAE	RMSE
no positional information		3.58	6.68
SOAP		4.74	6.99
aniAEV		3.93	6.09
EquiformerV2	omat	3.21	4.50
MACE	mace-mp0	2.44	4.09
	mace-off	2.46	4.17

Compared to the results without positional information, only the descriptors as used in the MACE and Equiformer architecture contribute positively to improving the predictive accuracy of the D-MPNN for activation energies. In contrast, the SOAP and aniAEV descriptors appear to introduce more noise as the MAE increases while the RMSE reduces slightly, indicating the removal of outliers. It appears that there is a difference in performance between the fixed (SOAP and aniAEV) and learned (MACE and Equiformer) descriptors, making the gained performance very sensitive to the details of the encoding of the 3D local environment. We hypothesize that learned descriptors provide a more useful description of the atomic local environment since their large-scale pretraining on QM energies and forces drives the descriptors toward a depiction of the wavefunction. In contrast, fixed descriptors merely encode the local geometric information using radial and spherical terms.

#### 3.2.2 Influence of reactant, product, and transition state positions on the prediction accuracy

As a next step, we investigated the incorporation of different combinations of coordinate information from the reactant (r), transition state (ts) and/or product (p). The inclusion of only the reactant or product information reduced the MAE and RMSE metrics marginally (MAE changed from 3.64 to 3.52 and 3.42, respectively). The same applied to including both reactant and product information (MAE of 3.46). A significant reduction in error metrics was achieved when transition state positions were incorporated. The MAE then varied between 2.29 and 2.38, and the RMSE between 3.75 and 3.80. Hyperparameter optimised values are shown in Table S3 in the SI.

#### 3.2.3 Comparing embedding strategies for positional information

Next, we tested different embedding strategies using the MACE (with mace-mp0 potential) descriptor. We tested different ways of embedding when employing all coordinates (r, ts, p) as well as when only using transition state coordinates (ts). In Table 3, the impact of the different embeddings on the model's performance is summarised. When coordinate features were incorporated after the MPNN, performance improved significantly. The pure inclusion of positional descriptors reduced the MAE to 2.65 and the RMSE to 4.18 (values labeled as ’plain’ in Table 3). Further enhancement with a ReLU activation function led to the lowest MAE in this group (2.52) and RMSE (3.87), indicating that nonlinearity improves the model's ability to extract meaningful features from the coordinate information. However, the addition of an attention layer did not yield further improvement. Incorporating coordinate features before message passing resulted in slightly better predictions compared to adding them after the MPNN. When only transition state coordinates were used, the trend remained consistent, suggesting that adding additional features before the MPNN using a linear layer plus a ReLU activation function provides the best overall performance.

**Table 3:** Comparison of different strategies for embedding structural information. Various methods are evaluated: direct concatenation (no additional), passing the additional features directly to the model (plain), passing them through a linear layer followed by a ReLU activation function (+ relu fkt) or passing them through a linear layer with a ReLU activation function combined with an attention layer (+ att layer). The impact of these strategies is assessed using MAE and RMSE values. The best values for using all available coordinate information (r, ts,p) and only the transition state (ts) are highlighted in bold. All values are in kcal mol^−1^

**Coordinates**	**Embedding**	**Layers**	**MAE**	**RMSE**
		no additional	3.58	6.68
r, ts, p	After MPNN	plain	2.65	4.18
		+ relu fkt	2.52	3.87
		+ att layer	2.56	4.13
	Before MPNN	plain	2.46	**4.01**
		+ relu fkt	**2.40**	4.12
		+ att layer	2.48	4.07
ts	After MPNN	plain	2.76	4.57
		+ relu fkt	2.72	4.26
		+ att layer	2.79	4.66
	Before MPNN	plain	2.43	4.12
		+ relu fkt	**2.40**	**4.04**
		+ att layer	2.50	4.10

### 3.3 On-the-fly prediction of 3D conformations

Despite the success of the above models, utilizing ground truth coordinates in a barrier height prediction model is meaningless, since energies are easy to obtain by quantum mechanics once the geometry is known. In the following, we therefore explore the on-the-fly prediction of coordinates on the RDB7 and RGD1 datasets. As outlined in the Methods section, TSDiff and GoFlow were trained on the ground truth geometries using the train and validation splits. We then train the D-MPNN model on the same train and validation splits, incorporating the ground truth geometries as atom features. For the final barrier height predictions (using the test set never seen in the TSDiff/GoFlow training), we augment the D-MPNN model with descriptors derived from transition state geometries generated on-the-fly by these models.

Table 4 summarizes the MAE and RMSE results without (no coord) and with coordinates either provided from QM calculations (ground truth coord), from TSDiff (diffusion coord) or from GoFlow (flow matching coord) following the procedure described before. As expected, models trained with access to ground truth coordinates achieve the lowest errors across all datasets, with substantial improvements compared to models without any coordinate information. The benefit of incorporating 3D information is particularly pronounced for the more challenging splits, such as the reaction core and barrier-height splits. Nevertheless, when using the diffusion-predicted coordinates from TSDiff, there is still a notable improvement compared to the no-coordinates baseline for the RDB7 dataset. The RMSE for RDB7 decreases from 6.68 to 5.97 while the MAE decreases from 3.58 to 3.27.

**Table 4:** Performance metrics (MAE and RMSE) without (no coord) and with coordinates from QM calculations (ground truth coord), from GoFlow (flow matching coord), or from TS-diffusion (diffusion coord). We included coordinate information only for the transition states by using a linear layer to learn the MACE-mp0 descriptor, concatenating it with the default feature vector before the MPNN. All values in kcal mol^−1^

Dataset	Split	No coord		Ground truth coord		Flow matching coord		Diffusion coord	
		MAE ± STD	RMSE ± STD	MAE ± STD	RMSE ± STD	MAE ± STD	RMSE ± STD	MAE ± STD	RMSE ± STD
RDB7	random	3.58 ± 0.08	6.68 ± 0.13	2.44 ± 0.08	4.07 ± 0.07	3.13 ± 0.04	5.74 ± 0.04	3.27 ± 0.04	5.97 ± 0.06
	reaction core	4.64 ± 0.14	7.17 ± 0.15	3.36 ± 0.03	5.10 ± 0.04	4.56 ± 0.07	7.26 ± 0.06		
	barrier height	21.41 ± 0.06	26.60 ± 0.03	16.84 ± 0.22	21.50 ± 0.26	19.35 ± 0.17	23.96 ± 0.11		
RGD1	random	4.02 ± 0.01	6.91 ± 0.03	2.07 ± 0.02	3.12 ± 0.02	3.86 ± 0.02	7.10 ± 0.03	5.27 ± 0.02	9.32 ± 0.03
	reaction core	4.83 ± 0.03	7.79 ± 0.02	2.58 ± 0.02	3.82 ± 0.02	4.31 ± 0.02	7.80 ± 0.03		
	barrier height	23.76 ± 0.24	32.96 ± 0.25	13.77 ± 0.16	24.80 ± 0.13	16.45 ± 0.01	27.57 ± 0.02		

For the RGD1 dataset, we obtained worse results when using diffusion coordinate information compared to having no coordinates at all. Analyzing the predicted geometries revealed unphysical geometries where two molecules are pushed far away from each other (an example is shown in Fig. S3). However, even removing the 482 worst outliers and only predicting barrier heights for the remaining 8365 reactions did not yield a better model performance compared to using no coordinates, thus suggesting that the diffusion model overall has problems predicting reasonable coordinates for this dataset.

Furthermore, we trained our recently introduced GoFlow model^30^ on the RDB7 and RGD1 datasets, which led to a substantial reduction in MAE for the investigated datasets. With the random split, the MAE for RDB7 improved from 3.58 to 3.13 (RMSE: 6.68 to 5.74), whereas for RGD1, it decreased from 4.02 to 3.86 (RMSE: 6.91 to 7.10). The harder reaction core and barrier height split, including 3D information from the flow matching model, led to reduced MAE and RMSE values as well. For the two examined datasets, we demonstrate that 3D coordinates predicted on-the-fly using GoFlow can enhance reaction barrier height predictions and outperform models with TSDiff coordinates. Yet, the performance gap between models with generated and ground truth coordinates indicates opportunities for further refinement of generative approaches.

To explore how the quality of training data affects predictions on the RDB7 dataset, we conducted a cross-validation study. Previously, we always trained models using the original QM coordinates, only varying the coordinates for the test set according to the method used (ground truth, flow matching, or diffusion-generated). This reflects the intended real-world scenario, as QM computations are typically required to obtain training activation energies, thereby providing suitable coordinates to train generative models. To gain deeper insights into the flow matching model, we performed a cross-validation analysis by splitting the entire RDB7 dataset into 20 folds, ensuring each reaction appeared exactly once in the test set. We then used the predicted coordinates to train our DMPNN for barrier height prediction. This approach yielded a slightly worse performance (MAE: 3.32, RMSE: 6.26) compared to the model trained with ground truth coordinates.

## 4 Conclusion

In this work, we explored strategies to improve the accuracy of predicting reaction barrier heights using D-MPNN models by incorporating either additional 2D information through ml-QM features or by including 3D structural information. We evaluated the utility of ml-QM descriptors, finding that they offer minor improvements, especially for small and chemically diverse datasets, while introducing minimal computational overhead.

Incorporating 3D structural information in the form of ground truth coordinates consistently reduced both MAE and RMSE across all evaluated datasets, demonstrating the strong potential of using geometric data in barrier height prediction. To eliminate the dependency on precomputed coordinates, we retrained the TSDiff and GoFlow models to generate transition state geometries directly from 2D inputs. Among these, GoFlow proved especially effective, yielding notable improvements in predictive accuracy. These results highlight the advantage of incorporating predicted 3D structures to enhance model performance, bridging the gap between 2D input and 3D informed output. However, while GoFlow brings performance closer to results achieved with ground truth geometries, a gap remains, indicating opportunities for further refinement of the generative models.

## Author contributions

JK: conceptualization, formal analysis, investigation, methodology, software, validation, visualization, writing – original draft. JDL: investigation, methodology, software, writing – review & editing. LG: data curation, formal analysis, investigation, methodology, software, writing – review & editing. TJ: methodology, investigation EH: conceptualization, funding acquisition, methodology, project administration, supervision, writing – review & editing.

## Conflicts of interest

There are no conflicts of interest to declare.

## Supplementary Material

DD-004-D5DD00240K-s001

## Data Availability

All datasets used in this study (RDB7 and RGD1), as well as the archived version of ChemTorch used for this study are available at https://zenodo.org/records/17104892. Additionally, data for RDB7, the specific ChemTorch version and instructions for reproducing the results are available at https://github.com/JohannesKarwou/chemtorch. Supplementary information is available. See DOI: https://doi.org/10.1039/d5dd00240k
