# Supplementary material for: Graph-based prediction of reaction barrier heights with on-the-fly prediction of transition states
Source: Digit Discov. 2025 Sep 15;4(11):3208–16. doi: 10.1039/d5dd00240k (PMC12461184; doi:10.1039/d5dd00240k)
Supplement: DD-004-D5DD00240K-s001 [file DD-004-D5DD00240K-s001.pdf]

Supplementary Information (SI) for Digital Discovery.
This journal is © The Royal Society of Chemistry 2025

Supplementary Information:

Graph-based prediction of reaction barrier heights with on-the-fly prediction of transition states

Johannes Karwounopoulos, Jasper De Landsheere, Leonard Galustian, Tobias Jechtl, and Esther Heid[a)]

Institute of Materials Chemistry, TU Wien, A-1060 Vienna, Austria

[a)]Electronic mail: esther.heid@tuwien.ac.at

A. Hyperparameter optimisation

In Table S1, the hyperparameters are listed that were used for the bayesian hyperparameter search.

**Parameter**	**Values**
learning_rate	[0.0001, 0.001, 0.002]
model.dropout	[0, 0.05, 0.1]
model.layer_cfg.dropout	[0, 0.05, 0.01, 0.1]
model.depth	[2, 4, 6]
model.hidden_channels	[600, 900, 1200]
model.head_cfg.dropout	[0, 0.02, 0.1]
model.head_cfg.hidden_channels	[100, 300, 600]
model.head_cfg.num_layers	[1, 3, 5]

Table S1. Hyperparameter values used in the Bayesian optimization search.

B. QM descriptors

In Table S2, the tested features for each category (atom, bond or molecular feature) are described.

Category	Feature	Description
Atom Features	NPA (Natural Population Analysis)	Summation over all NAOs of a given atom to obtain the Natural Charges for each atom.
	Fukui Indices	Predicts reactivity in three modes: f(+): nucleophilic sites, f(-): electrophilic sites, f(0): radical sites.
	Valence Orbital Occupancy	Represents the number of electrons occupying an atom's valence orbitals, influencing bonding and reactivity.
	NPA Charge	Net charge derived from NPA calculations.
	NPA Charge (+)	Positive mode NPA charge calculation.
	NPA Charge (-)	Negative mode NPA charge calculation.
	NPA Parr Function (+)	Positive Parr function for NPA, showing where nucleophilic attack is likely.
	NPA Parr Function (-)	Negative Parr function for NPA, indicating electrophilic attack sites.
	Shielding Constant	Constant describing the shielding effect of electrons around the nucleus.
	Valence Orbitals	Electron counts in valence orbitals: 1s, 2s, 2p, 3s, 3p, 4s, 4p.
Bond Features	Bond Order	Measure of bond strength between atoms.
	Bond Length	Distance between bonded atoms.
	Bonding Electrons	Number of electrons in the bonding region between atoms.
	Natural Ionicity	Degree of ionic character in a bond.
Molecular Features	Dipole Moment	Measure of molecular polarity.
	Traceless Quadrupole Moment	A component of molecular shape and charge distribution.
	HOMO/LUMO	Energies of the highest occupied molecular orbital (HOMO) and lowest unoccupied molecular orbital (LUMO).
	HOMO-3 to LUMO+3	Energies for molecular orbitals close to the HOMO and LUMO.
	Ionization Potential (IP)	Energy required to remove an electron.
	Electron Affinity (EA)	Energy released when an electron is added to the molecule.

Table S2. Overview of atomic, bond, and molecular features as proposed in literature.[1]

C. Feature importance analysis

Figure S1 shows the permutation feature importance across three datasets (E2, RDB7, RGD1), highlighting which features most influence model predictions by measuring the increase in MAE when individual features are permuted. Features with a negative value are thus making the predictions better, so the model can benefit from them.

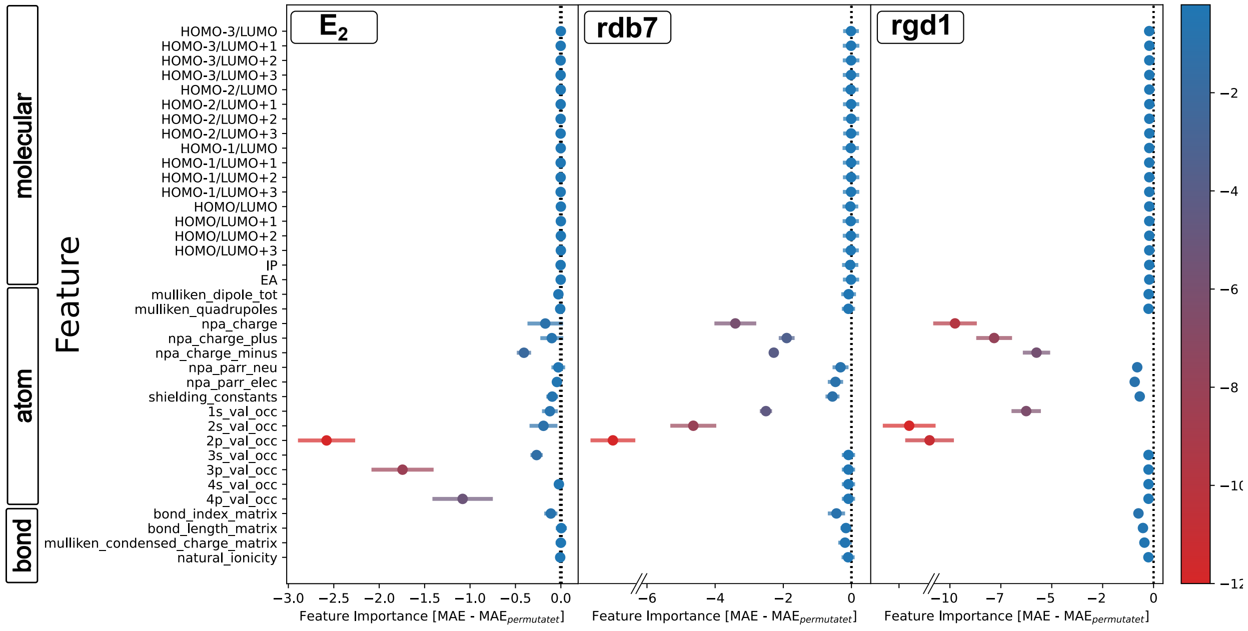

Figure S1. Permutation Feature Importance to assess the impact of individual features for three different datasets. The calculation follows these steps: Given a trained model $\hat{f}$, a feature matrix X, a target vector y, and the Mean Absolute Error (MAE) as the error measure, the original model error is first computed as $e_{\text{orig}} = \text{MAE}(y, \hat{f}(X))$. Then, for each feature j, its values in X are randomly permuted to generate a new feature matrix X_{perm}. The model error is recalculated using the permuted data as $e_{\text{perm}} = \text{MAE}(y, \hat{f}(X_{\text{perm}}))$. The feature importance score is then obtained as the difference $FI_j = e_{\text{perm}} - e_{\text{orig}}$.

Figure S2 compares the model performance (MAE) across eight datasets when trained on varying numbers of data points (400–1000), using different feature sets. It shows that incorporating additional molecular, atom, and bond features generally improves performance over default features, with the combined use of all features yielding the lowest MAE across most datasets.

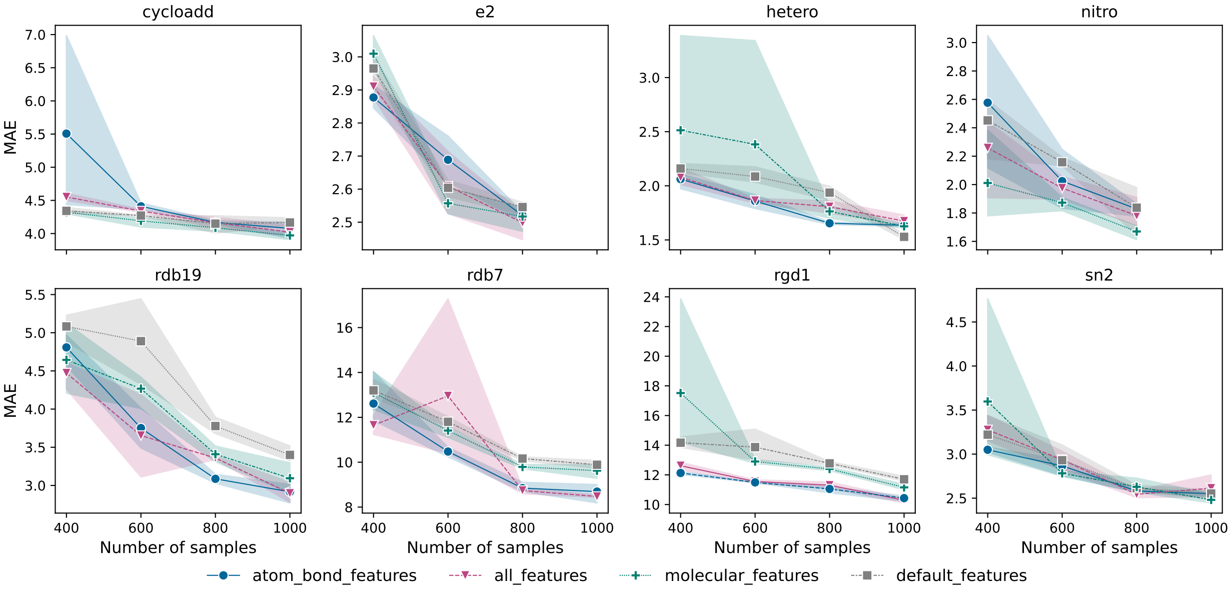

Figure S2. Evaluation of the mean absolute error (MAE) across the investigated datasets using only 400, 600, 800 or 1000 data points of the respective dataset. The dots represent the average values computed over three runs with different random seeds, while the shaded region indicates the error bars. Results obtained using the default features are shown in gray, those incorporating additional molecular features are displayed in green, additional atom and bond features are also represented in green, and the results utilizing all features are shown in pink.

D. Influence of reactant, product, and transition state positions on the prediction accuracy

In Table S3 the influence of including positions from eiter reactant, product, transition state or a combination of it on the MAE and RMSE is shown.

**Including positions from:**	**MAE**	**RMSE**
no positions	**3.58 ± 0.08**	**6.68 ± 0.13**
r	3.52 ± 0.10	6.52 ± 0.09
r, p	3.46 ± 0.01	6.31 ± 0.10
p	3.42 ± 0.04	6.13 ± 0.05
ts	2.37 ± 0.03	3.80 ± 0.14
r, ts	2.29 ± 0.06	3.75 ± 0.08
r, ts, p	2.38 ± 0.04	3.75 ± 0.06

Table S3. Test MAE and test RMSE for incorporating different positional information without any previous hyperparameter optimization.

E. 3D predictions from the Diffusion model

In Figure S3, the structure of one compound of the RGD1 dataset is shown, where the structure predicted by the diffusion model shows the highest Root Mean Square Deviation from the ground truth structure.

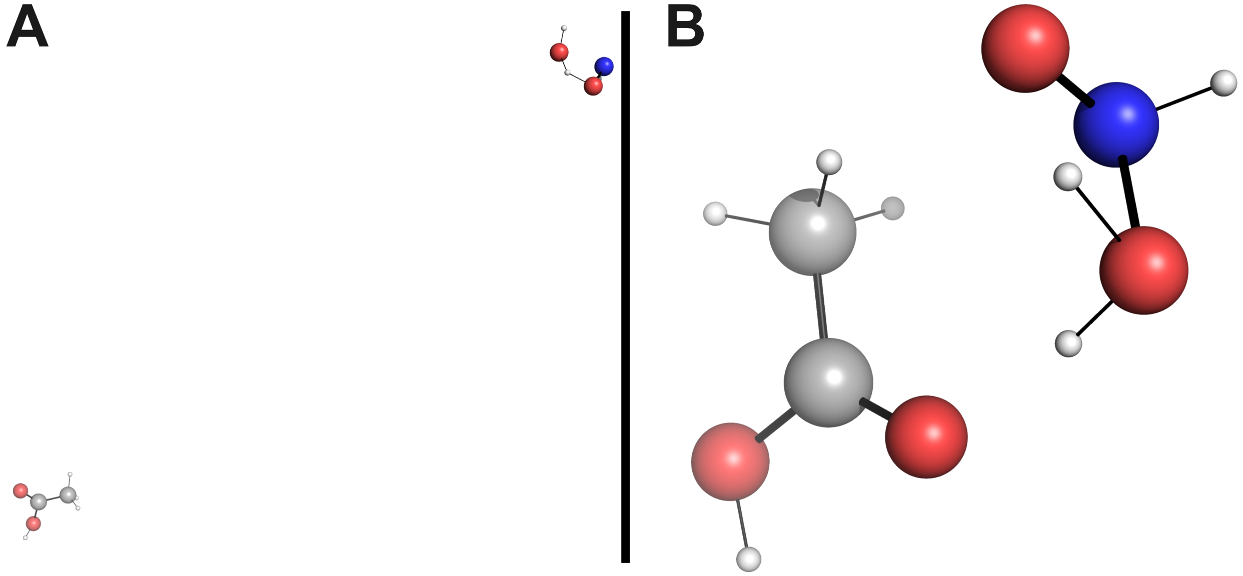

Figure S3. The molecule with the highest Root Mean Square Deviation between the coordinates generated by TSDiff (**A**) and the QM ground truth coordinates (**B**) for a compound from the RGD1 dataset (ID: 5655).

REFERENCES

[1]S.-C. Li, H. Wu, A. Menon, K. A. Spiekermann, Y.-P. Li, and W. H. Green, Journal of the American Chemical Society **146**, 23103 (2024), publisher: American Chemical Society.
